# Laser optomechanics

**DOI:** 10.1038/srep13700

**Published:** 2015-09-03

**Authors:** Weijian Yang, Stephen Adair Gerke, Kar Wei Ng, Yi Rao, Christopher Chase, Connie J. Chang-Hasnain

**Affiliations:** 1 Department of Electrical Engineering and Computer Sciences, University of California, Berkeley, CA 94720, USA; 2 Bandwidth10, Inc., San Jose, CA 95132, USA

## Abstract

Cavity optomechanics explores the interaction between optical field and mechanical motion. So far, this interaction has relied on the detuning between a passive optical resonator and an external pump laser. Here, we report a new scheme with mutual coupling between a mechanical oscillator supporting the mirror of a laser and the optical field generated by the laser itself. The optically active cavity greatly enhances the light-matter energy transfer. In this work, we use an electrically-pumped vertical-cavity surface-emitting laser (VCSEL) with an ultra-light-weight (130 pg) high-contrast-grating (HCG) mirror, whose reflectivity spectrum is designed to facilitate strong optomechanical coupling, to demonstrate optomechanically-induced regenerative oscillation of the laser optomechanical cavity. We observe >550 nm self-oscillation amplitude of the micromechanical oscillator, two to three orders of magnitude larger than typical, and correspondingly a 23 nm laser wavelength sweep. In addition to its immediate applications as a high-speed wavelength-swept source, this scheme also offers a new approach for integrated on-chip sensors.

The coupling of an optical field and a mechanical oscillator through forces induced by light has been a subject of long-standing interest and an important tool to study fundamental physics^1^,^2^,^3^,^4^,^5^,^6^. Numerous recent experiments have used light-induced forces in optical cavities to demonstrate phenomena including laser cooling^7^,^8^,^9^,^10^,^11^ and regenerative oscillation^12^,^13^,^14^,^15^,^16^,^17^, with applications such as quantum entanglement of macroscopic objects^18^, ultrasensitive measurement of displacements and forces^19^, and all-optical RF signal processing^20^. The prevalent experiment apparatus consists of a passive optical cavity housing a mechanical oscillator with a population of circulating photons produced by an external low-noise continuous-wave laser. In such setups, light can induce forces on a mechanical resonator directly through radiation pressure or indirectly through photothermal forces, which result from thermal expansion of the structure under light-induced heating. Despite recent progress, regenerative mechanical oscillation has been typically limited to nanometer-scale amplitudes^12^,^13^,^14^,^15^,^16^,^17^ for oscillators using radiation pressure and limited to low-kHz-scale frequencies for oscillators using photothermal effects^21^. Here, we show a new configuration that integrates the mechanical oscillator as a mirror in a laser cavity, as shown in Fig. 1, producing both high-speed and large-amplitude regenerative oscillation. The difference from conventional cavity optomechanics is that the cavity contains optical gain, serving as the laser itself to drive the mechanical oscillator and thus integrating an optomechanical system in a single device. The exploration of active optomechanics has been primarily limited to theoretical studies of optomechanical cooling^22^. While one experimental work has studied active optomechanics phenomena in an optically-pumped VCSEL cavity with monolithic, fixed distributed Bragg reflectors (DBRs), it primarily demonstrated the influence of externally-injected phonon pulses on the optical cavity^23^. Sustained mechanical self-oscillation in a lasing cavity has not yet been demonstrated.

Collocating a VCSEL cavity and the mechanical oscillator has multiple functions. Firstly, the mechanical oscillation of the mirror results in periodic oscillations of both the laser wavelength *λ* and output power *P*, as shown in Fig. 1. Since the VCSEL supports a single on-resonance longitudinal mode over a wide range of wavelength, the optomechanical forces, whether radiation-pressure or bolometric forces, can stay enhanced over a broad wavelength range. Secondly, the negative damping induced by optomechanical forces is greatly strengthened by the delayed response of the optomechanical forces to the mechanical movement of the mirror, which can be attributed to the long lifetime of the excited states in the gain medium. Furthermore, the top mirror reflectivity can be selected to have a strong dependence on the lasing wavelength, which is determined by the mirror displacement. Engineering the HCG reflectivity spectrum can thus enable design for devices exhibiting various phenomena, such as extremely large oscillation amplitude or cooling of the mirror’s mechanical modes. Self-oscillation using this scheme can thus be implemented in any mechanically-tunable laser possessing a mirror with wavelength-dependent reflectivity and a mechanical tuning system with high quality factor. The new scheme facilitates a strong power coupling between the optical radiation field and the mechanical oscillator. We term it laser optomechanics.

An optomechanical laser naturally acts as a wide-range high-speed self-wavelength-swept light source. This has been long sought for high-speed energy-efficient 3D imaging, such as optical coherence tomography^24^ and light detection and ranging^25^. Laser optomechanics immediately can address the demand for improved light sources in these fields. Additionally, replacing the external high-power laser source with a low-power, microscale VCSEL provides an advantage in existing applications of optomechanical self-oscillation, particularly on-chip RF clock generation^26^.

The optomechanical laser cavity demonstrated here uses an ultra-light weight (130 pg) high-contrast-grating (HCG) vertical-cavity surface-emitting laser (VCSEL)^27^,^28^,^29^ electrically-pumped lasing at 1550 nm. The HCG is an InP grating with near-wavelength period, designed to reflect >99.5% surface-normal light and serve as the top mirror of the VCSEL^30^,^31^. The HCG platform offers substantial design flexibility. It can be designed such that the high reflection is selected for light polarized in parallel or in perpendicular to the grating bar. The former is called TE HCG, while the latter TM HCG. The optimal dimensions are quite different, particularly in thickness. For 1550 nm operation, the TE HCG is typically ~195 nm thick and the TM HCG is ~430 nm thick. In this paper, we show that lasers of both TE and TM designs exhibit laser optomechanical self-oscillation. Detailed device designs can be found in Reference 29. Additionally, the ultra-small size and thin, long support arms of the HCG mirror allow its temperature to vary significantly from that of the bulk of the chip, enabling photothermal distortion forces. As a result, the optomechanical forces described in this work include contributions from both radiation pressure and photothermal forces. The rest of the VCSEL cavity contains an active region with multiple quantum well and a distributed Bragg reflector (DBR) as the bottom mirror. Current confinement is provided with a wide-aperture proton implant, ensuring a low beam divergence angle of <5 degrees^32^. In ambient temperature and pressure, the HCG-VCSEL lases with a DC threshold current of 5 mA and emits 1.7 mW at 12 mA bias with a single, fundamental transverse mode. The lasing wavelength of the VCSEL can be tuned by electrostatically displacing the HCG with a voltage across the HCG layer and the sacrificial layer, which changes the cavity length. Figure 2a shows the schematics of the HCG VCSEL, and Fig. 2c shows the scanning microscope image (SEM) of a TE HCG. The HCG mirror is 20 μm × 20 μm in size and 195 nm thick, with a total weight of only ~130 pg, two orders of magnitude lighter than a standard DBR mirror. The mirror is held by a double bridge spring structure. Under vacuum, this micro-electro-mechanical (MEMS) structure exhibits a high mechanical quality factor *Q*_*m*_, which can be measured by Laser Doppler vibrometry (LDV) (see Methods for details). Figure 2b shows LDV characterization of a fundamental-mode mechanical resonance of a TE-type device under small-signal electrostatic white noise actuation, fitted with a Lorentzian function showing a quality factor *Q*_*m*_ = 3,640 at a frequency of 147 kHz. It is important to note that the VCSEL cavity is not pumped, and the wavelength of the external laser used in the LDV measurement is far from the VCSEL cavity resonance. Thus, LDV directly measures the mechanical property of the MEMS HCG unperturbed by the VCSEL’s optomechanical effects.

The HCG self-oscillates at its mechanical resonance frequency with the VCSEL biased above threshold with 9 mA DC current in a vacuum of 2e–5 Torr. The self-oscillation of the HCG can be visualized by the SEM images of the HCG self-oscillating at fundamental mechanical mode, shown in Fig. 2d with 13 mA DC currents. A fast scanning rate is used so that stroboscopic effect can be visualized on the grating bars, from which the oscillation frequency and amplitude are calculated to be ~131 kHz and ~595 nm (see Supplementary Information). This measured amplitude is two to three orders of magnitude larger than those typically reported in optomechanical oscillators^12^,^13^,^14^,^15^,^16^,^17^. Compared with other MEMS-tunable lasers, the advantage of the HCG VCSEL is a combination of its light weight and high mechanical quality factor, which together reduce damping forces on the mirror, allowing optomechanical dynamics to more readily produce self-oscillation (detailed analysis in Supplementary Information).

In laser optomechanics, the laser emission wavelength oscillates as the cavity mirror position oscillates. Figure 3a shows the results of time-resolved characterization for a typical TE HCG VCSEL undergoing optomechanical self-oscillation (see Methods for details). Simultaneously, the optical spectrum of the device was captured with an optical spectrum analyzer, as shown in Fig. 3b, confirming the total wavelength excursion of 23 nm results from the HCG oscillation with fundamental mechanical mode. Considering an intrinsic linewidth of 50 MHz for a typical HCG VCSEL^29^, the 23 nm wavelength shift corresponds to 57,500 times the intrinsic linewidth. Due to the longitudinal profile of the VCSEL mode, this wavelength shift implies a 560 nm vertical oscillation amplitude, confirming the separate SEM measurements described previously. The wavelength oscillation is accompanied by an oscillation of the optical power from the VCSEL. At the two ends of the oscillation excursion, the laser stops lasing. Thus the actual mechanical oscillation amplitude is larger. The circulating optical power in the laser cavity depends on mirror position both directly, through mechanically-induced deformation in the mirror, and indirectly, via the wavelength-dependent laser gain spectrum and mirror reflectivity spectrum. Under static conditions, the optomechanical forces act on the mirror proportionally to the circulating optical power in an optical spring effect, but when retarded by the dynamics of the laser cavity, the position-dependent optomechanical forces create instability the mechanical oscillator, leading to optomechanical self-oscillation. During large-amplitude self-oscillation, mechanically nonlinear effects produce nonharmonic components, as are visible in the electrical power spectral densities in Fig. 4a,d.

In conventional cavity optomechanics, optical input power and detuning are the two control knobs to tune the optomechanical dynamics^33^; in laser optomechanics, these correspond to the laser pumping strength and the cavity length, and specifically, the laser drive current and the tuning voltage of the HCG MEM structure. The laser current primarily changes the circulating power and the photon retardation (see Supplementary Information for detailed analysis), whereas the tuning voltage mainly modifies the spectral overlap between the gain spectrum and cavity resonances and thus the gradient of the optomechanical forces versus the HCG displacement. Figure 3c shows an example of this control. The optical wavelength swept range is measured with various laser currents and tuning voltages. Pump current increases the quantum well optical gain and thus the optomechanical damping, leading to a larger wavelength sweep. As the tuning voltage increases and cavity length shortens, the wavelength sweep is observed to decrease. This is most likely attributable to a reduced optomechanical anti-damping due to lower mirror reflectivity or decreased optical gain from the quantum wells at short wavelengths. Most importantly, this experiment suggests that the opposite effect, mechanical cooling, can be realized in laser optomechanics as well with careful engineering of the laser parameters and the mechanical structure.

In laser optomechanics, various mechanical modes can be excited. The mechanical damping factor and the gain from optomechanical forces determine the oscillation threshold. The modes with the lowest threshold will be first excited, analogously to optical lasing. Figure 4 shows two different states of a VCSEL with a TM HCG: the pure fundamental mode at 345.6 kHz and a pure high order mode at 5.636 MHz. The TM HCG in this device has a size of 20 μm × 20 μm, and a thickness of 430 nm — thicker than the TE HCG. The different states can be manipulated by the pump current and tuning voltage. When oscillating at high order mode, the 3 dB linewidth of the fundamental radio-frequency (RF) tone is 8 Hz, leading to a loaded mechanical quality factor of 7 × 10^5^. It is interesting to see there appear multiple RF tones (Fig. 4e). This mode splitting of ~44 Hz could be attributed to the optical mode having a Gaussian spatial profile that couples slightly differently to each grating bar. At certain values of DC laser current and tuning voltage, both modes are excited, and have been observed in self-oscillation simultaneously (see Supplementary Information). These interesting phenomena show the rich physics to be further explored in laser optomechanics.

The mechanical modes of the HCG can be mainly divided into three categories, represented by Fig. 5a–c. Figure 5a depicts the fundamental mode, where the whole HCG moves in and out of its plane vertically. Figure 5b,c depict examples of the higher order mechanical modes, which consist of oscillations of the HCG frame and bars in 5b, and of just the HCG bars in 5c. For simplicity, we analyze only the fundamental mechanical mode with vertical position coordinate *x*(*t*), modal mass *m*, resonant angular frequency ω_o_, and damping rate *b*. This analysis can be readily extended to multimode oscillation including modes of different generalized position coordinates, masses, and frequencies in a larger differential equation system. The oscillator mode is acted upon by the optomechanical forces *F*_*OM*_, which comprises the radiation pressure force *F*_*RP*_ and the photothermal force *F*_*PT*_, the latter of which acts by expanding the grating bars or support arms of the HCG mirror under heating. The position *x*(*t*) obeys a driven damped harmonic oscillation:
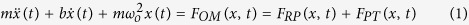


While both force terms are a function of both the mirror displacement *x* and time *t*, they represent delayed responses to the mirror position, and thus can be written as 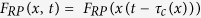 and 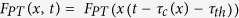. The delay *τ*_*c*_ is the retardation between a change in *x* and change in optical circulating power inside the cavity (and thus radiation pressure force *F*_RP_), and is based upon the effective photon lifetime of the active cavity. The photothermal force is further delayed by *τ*_*th*_, the thermal response time constant of the HCG. Compared to the mechanical oscillation period of 100 ns to 10 μs, *τ*_*c*_ is much smaller, generally from 100 ps to 1 ns in a VCSEL cavity. While this delay can be approximated as small compared to the oscillation period, it is necessary to explain the anti-damping effect of optomechanical forces. If the thermal transient time of the HCG were comparable to the oscillation period, a lowpass term would be added to its delay expression (see Supplementary Information for details). With Taylor expansion, both *F* can be written as 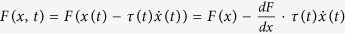. Equation (1) can be re-arranged as:
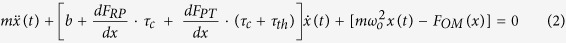


Equation (2) clearly reveals two functionalities of the optomechanical forces. Firstly, it changes the oscillation frequency, which can be described as the optical spring effect^34^. Secondly, an optomechanical damping term of form 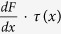 is added onto the original mechanical damping *b* for each one of the optomechanical forces. With this in mind, the above equation can be rewritten
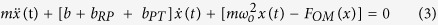
where *b*_*RP*_ and *b*_*PT*_ represent radiation pressure and photothermal damping. When *b*_*RP*_ + *b*_*PT*_ < 0, optomechanical forces act as a mechanical gain, and if this anti-damping cancels the mechanical damping, regenerative oscillation can occur. Analysis of the laser rate equations can provide *τ*_*c*_(*x*) and 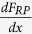, while 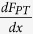 and *τ*_*th*_ additionally require measurements or simulation of the device’s thermal expansion under photoabsorption (see detailed analysis in the Supplementary Information). While photothermal forces are significantly smaller than radiation pressure in this high-reflectivity and low-absorption HCG mirror, the large thermal delay *τ*_*th*_ can make the net optomechanical gain, and thus power transfer, of photothermal effects comparable to radiation pressure in this device.

Compared to passive cavities, active optomechanical cavities offer optomechanical gain *b*_*RP*_ + *b*_*PT*_ < 0 over a wider position range, sustaining large-amplitude oscillation. Figure 5d–e presents a comparison between active-cavity and passive-cavity radiation-pressure optomechanics based on the locally linearized responses of the radiation pressure force to changes in mirror displacement. This compares *τ*_*c*_(*x*) and *b*_*RP*_ for an active optomechanical cavity and a comparable passive optomechanical cavity. For a general comparison, the passive cavity is simulated with an input power equaling that of the output power of the active cavity (2 mW), while its optical quality factor *Q*_*p*_ = 17,600 equals that of the active cavity considering only the mirror-loss component. The active cavity has a total quality factor *Q*_*a*_ = 8,800 including internal absorption (see Supplementary Information for details). In this comparison, laser optomechanics shows a greatly enhanced *τ*_*c*_(*x*) (>10X) in a much broader wavelength range (>100X) than the passive optomechanical cavity. This is attributed to the long carrier life time of the gain medium, due to large reservoir of carriers staying in the excited states. This serves as the buffer for the circulating photons inside the cavity, and effectively increases the photon lifetime. In the passive cavity, on the other hand, there is no such extra buffer, and the photon lifetime is solely determined by the passive cavity. Therefore, a very high-Q cavity is generally desired in passive optomechanical cavities to show strong interaction between the light field and the mechanical oscillator^6^. Active optomechanical cavities thus provide an alternative path to slow the cavity response time and effectively increase the cavity Q-factor. Another unique property of laser optomechanics is that the high optical Q-factor as well as the large optical circulating power can be maintained over a large mirror displacement, as the lasing wavelength is locked with the cavity. In the regenerative oscillation regime, the oscillation amplitude is ultimately determined by the nonlinearity and the gain spectrum of the laser. Furthermore, there is a great flexibility in designing the wavelength dependence of the mirror reflectivity and gain in laser optomechanics, which largely controls the 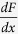 term in Eq. (2) (See Supplementary Information for details). This could be the key to further exploration of optomechanical dynamics. We note that the region of positive optomechanical damping in Fig. 5d suggests the possibility of self-cooling in active cavities.

The HCG VCSEL provides advantages over the conventional MEMS-tunable VCSELs with DBR as top mirrors. First of all, the reflection spectrum of the HCG can be engineered into various shapes, opening new possibilities in laser optomechanics. Secondly, since the mechanical damping factor 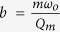 should be as small as possible for optomechanical dynamics, the HCG VCSEL provides a unique advantage due to its two-order of magnitude lighter weight compared to the DBR mirrors of conventional MEMS-tunable VCSELs^35^. Lastly, laser optomechanics with ultra-light HCG mirrors enables optomechanical coupling through both radiation pressure and photothermal forces, as shown in this work, offering large power transfer to the mechanical mode and an additional degree of configurability in designing optomechanical structures. For example, the use of bi-material structures or higher-absorptive materials in the HCG and its supports could further enhance photothermal effects above radiation pressure. While FEM simulations and dynamical analysis indicate that the large majority of optomechanical power transfer in the current device occurs due to radiation pressure and not photothermal forces (see Supplementary Information), the exact ratio remains a subject for future work.

To conclude, we have demonstrated a new paradigm of laser optomechanics using a VCSEL with an ultralight HCG mirror. A strong power transfer between photons and a mechanical resonator is achieved by collocating a laser cavity with a mechanical oscillator. We show that three key criteria are necessary to achieve high-amplitude self-oscillation: (1) an ultra-low-mass mechanical oscillator with a high mechanical quality factor, (2) a strong wavelength dependence of the mirror reflectivity, and (3) a sufficient delay from the laser rate equations due to long photon lifetime (optical Q) and long carrier lifetime in the active region. Such a laser optomechanical system will extend the functionality of tunable lasers by creating a high-speed wide-range wavelength-swept source for applications in optical coherence tomography (OCT), light detection and ranging (LIDAR) and 3D cameras, and will establish a platform to further study laser optomechanics.

## Methods summary

The 1550 nm HCG VCSEL is fabricated on an InP epitaxial wafer with AlGaInAs as the quantum wells. The HCG is defined by electron beam lithography followed by a wet chemical etch. The current aperture is defined by proton implantation. The HCG is released by selective wet chemical etch. See Ref. 29 for a detailed fabrication flow.

The mechanical quality factor of the MEMS HCG is measured independently from the optomechanical effects with a LDV system (Polytec OFV-3001). The HCG VCSEL is placed under vacuum to reduce the squeeze-film mechanical damping of the HCG in air. This technique resolves the velocity of a moving MEMS device using the Doppler frequency shift of the reflected beam from an external laser beam onto the device. Since the laser optomechanical cavity is not pumped and the external laser wavelength is far from the cavity resonance, the measurement will not be perturbed by the device’s optomechanical effects.

To characterize the optomechanical properties of the HCG VCSEL, the VCSEL is placed in a vacuum chamber, and a multimode cleaved fiber is used to couple the VCSEL output light into the fiber. The optical power is then split into three paths. One path is connected to a photodetector with 1.2 GHz 3-dB bandwidth. Another path is connected to a monochromator followed by a photodetector with the same bandwidth. The outputs of the two photodetectors are connected to power amplifiers with 250 MHz 3-dB bandwidth, followed by a real-time oscilloscope, for time-resolved optical power and lasing wavelength measurement. The pass-band of the monochromator is scanned across the wavelength swept range of the VCSEL, and the time-resolved lasing wavelength can be extracted from the series of traces obtained in the oscilloscope. Alternatively, the output of the amplifier can be connected to a RF spectrum analyzer to identify the mechanical modes and their properties. The third path of the split optical light is connected to an optical spectrum analyzer to characterize its average optical spectrum.

## Additional Information

**How to cite this article**: Yang, W. *et al.* Laser optomechanics. *Sci. Rep.* **5**, 13700; doi: 10.1038/srep13700 (2015).

## Supplementary Material

Supplementary Information

## Figures and Tables

**Figure 1:**
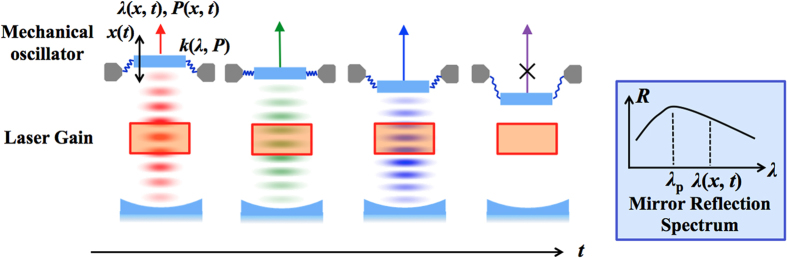
Schematic of experimental apparatus of laser optomechanics. The optical cavity houses the laser source as well as a mechanically moveable mirror with a reflection spectrum *R*(*λ*). The laser can be either optically pumped or electrically pumped. As the mirror oscillates with displacement *x*(*t*), the laser output wavelength *λ*(*x, t*) and power *P*(λ, *t*) changes with respect to time *t*. The spring constant *k* is a function of the optical wavelength and laser output power, which illustrate the optical spring effect. The various images visualize this wavelength changes: the lasing wavelength changes from red to blue as the effective cavity length shortens due to the mirror oscillation. Depending on the mirror reflectivity and gain, the mechanical oscillation can be sustained over a large displacement range.

**Figure 2:**
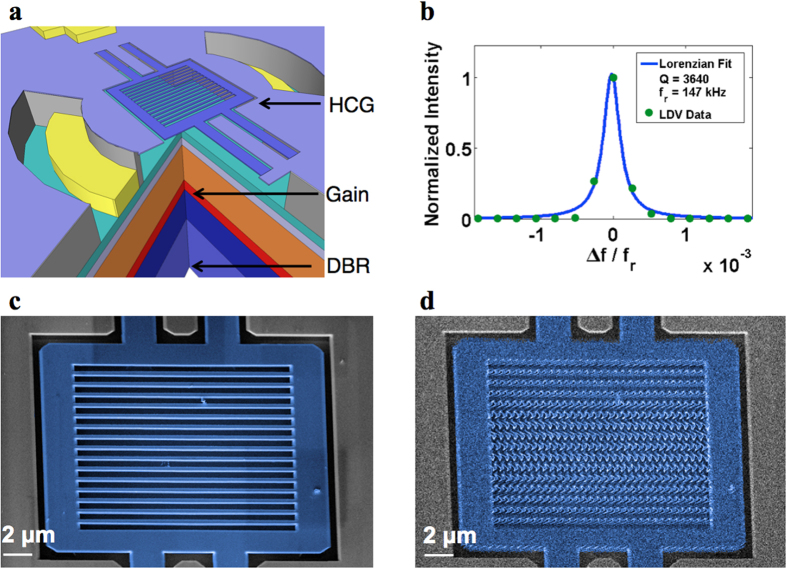
HCG VCSEL and the mechanical oscillation of HCG. (**a**) Schematic of the HCG VCSEL, which is composed of a capacitatively-actuated MEMS HCG top mirror, an active region with multiple quantum wells, and an immobile DBR bottom mirror. (**b**) Laser Doppler vibrometry measurement of quality factor of fundamental mechanical mode of MEMS oscillation in vacuum, *Q*_*m*_ = 3,640. (**c–d**) False color SEM image of the HCG viewed at 45^o^ from surface normal. **c** shows the steady HCG when the bias current *I* of the VCSEL is zero; (**d**) shows its oscillation at fundamental mode when *I* *=* 13 mA (laser threshold current is 5 mA at room temperature for this device). Due to the fast scanning rate of the SEM, the stroboscopic effect produces a periodic distortion of the HCG bars in the SEM image.

**Figure 3:**
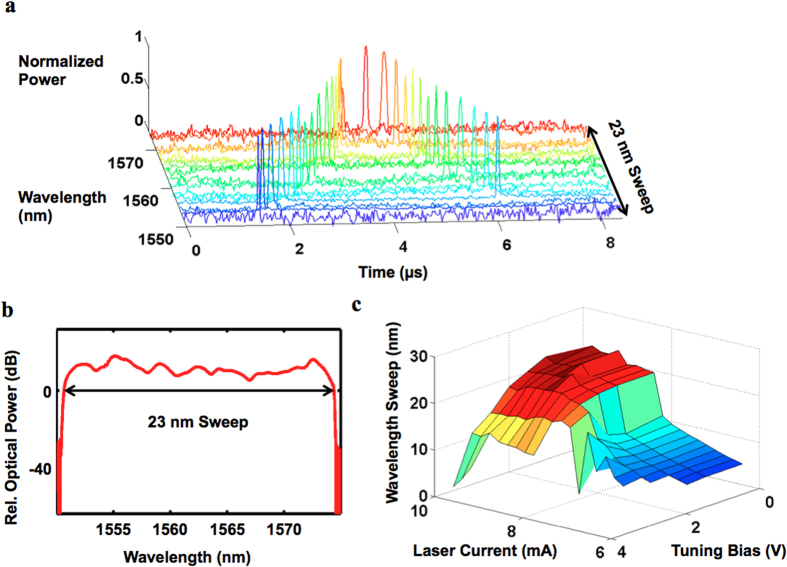
Regenerative oscillation of HCG VCSEL. (**a**) The time-resolved lasing wavelength of the HCG VCSEL with the HCG oscillating at fundamental mode. (**b**) Optical spectrum obtained in an optical spectrum analyzer with high integration time, captured simultaneously with the time-resolved wavelength characterization of Fig. 3a, confirming the extent of the wavelength sweep. (**c**) The wavelength swept range versus the laser current and tuning voltage. The laser current and tuning voltage can be used to tune the optomechanical dynamics.

**Figure 4:**
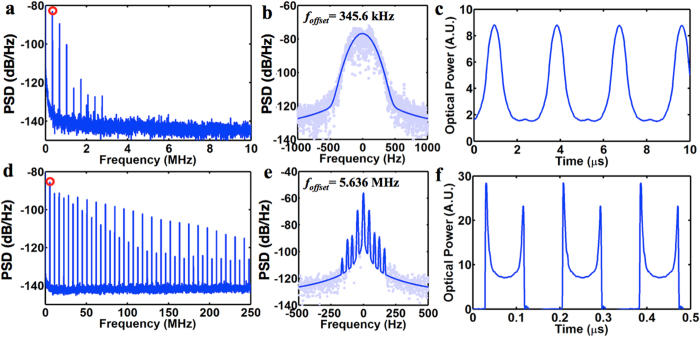
Regenerative oscillation of the HCG VCSEL in different mechanical oscillation modes. (**a–c)** Oscillation at fundamental mode; (**d–f**), Oscillation at a high order mode. (**a,d**) shows the RF spectrum density of the laser optical output power. (**b**,**e**) shows the zoom-in view of the first RF tone in (**a**,**d**) (marked with the red dot); they are fitted with Voigt functions. The frequency offset in (**b**) and (**e**) is 345.6 kHz and 5.636 MHz respectively. (**c,f**) shows the laser optical output power in time-domain. The threshold current of the laser is 10.1 mA at room temperature. The laser current and tuning voltage in conditions (a-c) and (d-f) is 19.2 mA, 10 V, 12 mA, 0 V respectively.

**Figure 5:**
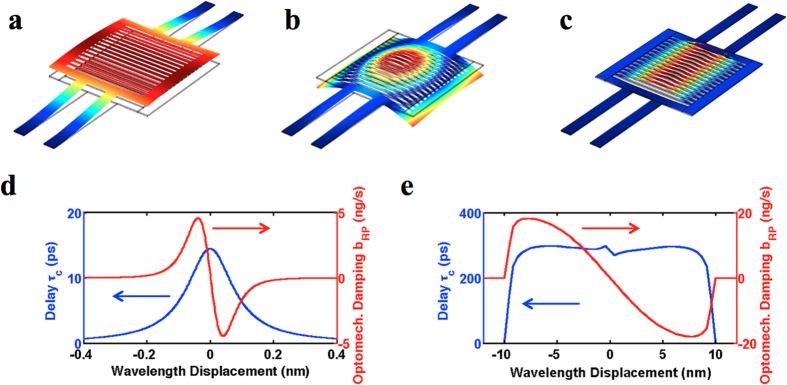
Mechanical modes of the HCG, and the analysis of the dynamical back action. (**a–c**), The mechanical modes of the HCG. **a** shows the fundamental mode, and (**b–c)** shows examples of the high order modes, where the HCG frame and bars are oscillating with the static bridges in (**b)** and only the HCG bars are oscillating with the static frame and bridges in (**c**). (**d**), Optical delay *τ*_*c*_(*x*) and radiation pressure damping 
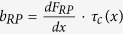
 versus wavelength displacement for a radiation-pressure passive optomechanical cavity. (**e**) Optical delay *τ*_*c*_(*x*) and radiation pressure damping 
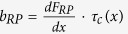
versus wavelength displacement for an equivalent radiation-pressure laser optomechanical cavity.
