# Supplementary material for: Laser optomechanics
Source: Sci Rep. 2015 Sep 3;5:13700. doi: 10.1038/srep13700 (PMC4558576; doi:10.1038/srep13700)
Supplement: Supplementary Information [file srep13700-s1.pdf]

Laser optomechanics

Weijian Yang[1], S. Adair Gerke[1], Kar Wei Ng[1], Yi Rao[2], Christopher Chase[2] & Connie J. Chang-Hasnain[1]

[1] Department of Electrical Engineering and Computer Sciences, University of California, Berkeley, CA 94720, USA
[2] Bandwidth10, Inc., San Jose, CA 94063, USA

SUPPLEMENTARY INFORMATION

I. HCG VCSEL cavity design

The 1550 nm HCG VCSEL is epitaxially grown on an InP substrate. Its cavity consists of an InP HCG as top mirror, an active region with multiple quantum wells made of AlGaInAs, and a DBR stack composed of InP/AlGaInAs layers. In between the HCG and the active region lie an air layer and two pairs of rigid InP/AlGaInAs DBR, which are used to enhance the overall top mirror reflectivity. The HCG is fully suspended in air. The HCG and two-pair DBR together form a compound mirror. Although the HCG can provide enough reflectivity to achieve lasing, the two-pair DBR provides tolerance towards surface roughness in the process of forming the freely suspended HCG. It also provides extra current spreading and is a standard design[29]. The HCG can be designed such that it is highly reflective towards the light with the polarization along the grating bars or perpendicular to the grating bars. The former one is referred as TE design, whereas the latter one TM design. For TE design, the HCG thickness, grating period and bar width is 195 nm, ~ 1050 nm and ~ 360 nm; for TM design, these values are 430 nm, ~700 nm, and ~ 450 nm. Their reflectivity is >0.995 at the wavelength of 1550 nm.

The HCG can be actuated towards the semiconductor layer by applying a voltage in between. This changes the air layer thickness and thus the lasing wavelength. A transfer matrix method is used to calculate the relationship between the lasing wavelength λ and the HCG displacement x, shown in Fig. S1. At the zero mirror displacement, the tuning efficiency $d\lambda/dx = 0.041$, corresponding to the optomechanical coupling coefficient $d\omega/dx = 5.12*2\pi$ GHz/nm, where ω is the angular frequency of the laser. The tuning efficiency, and thus the optomechanical coupling, are reduced compared to that of a Fabry-Perot cavity of comparable length because the two-pair top DBR reduces the fraction of the laser intensity incident on the HCG, the movable part of this compound reflector.

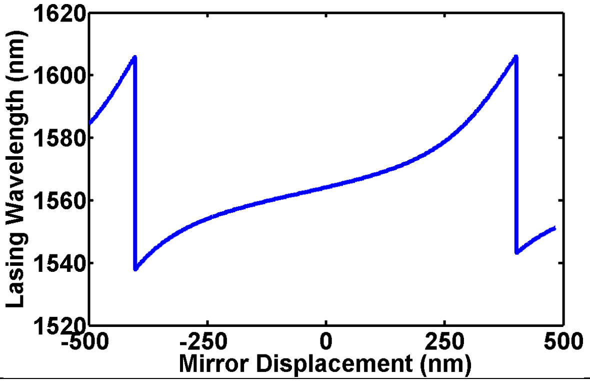

Figure S1 | The relationship between HCG displacement and the lasing wavelength in an HCG VCSEL. At the zero mirror displacement, dλ/dx=0.041, corresponding to the optomechanical coupling coefficient dω/dx = 5.12*2π GHz/nm.

The relationship between the tuning voltage V and the mirror displacement x can be calculated with a parallel capacitor transducer model. In general, we have the following formulation,

$$F_{spring} = k(d - d_0) \tag{S1}$$

where d_0 is the initial gap distance between the two plates (i.e. HCG and the substrate). When there is an actuation voltage V between the two plates, the gap distance becomes d. The two plates form a capacitor C. With an actuation voltage V, the energy W_e stored in the capacitor is

$$W_e = \frac{1}{2}CV^2 = \frac{\varepsilon_0 A}{2d}V^2 \tag{S2}$$

where A is the area of the HCG mirror and ε_0 is the dielectric permittivity in vacuum.

Thus the electrostatic force is

$$F_e = \frac{\partial W_e}{\partial d} = -\frac{\varepsilon_0 A}{2d^2}V^2 \tag{S3}$$

At the steady state $F_{spring} = F_e$, and thus we have,

$$(d - d_0)d^2 = -\frac{\varepsilon_0 A}{2k}V^2 \tag{S4}$$

With Eq. S4 and Fig. S1, the relationship between tuning voltage V and lasing wavelength λ can be obtained. Fig. S2 shows the theoretical calculation and the experimentally measured result, which are in a good agreement with each other.

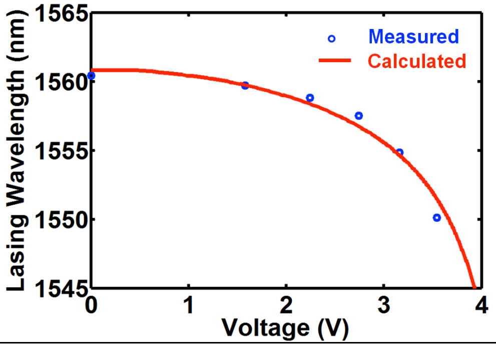

Figure S2 | The relationship between tuning voltage and the lasing wavelength in a TE HCG VCSEL. The blue dots show the experimental measured result. It agrees well with the theoretical calculation, shown as the red curve.

II. Mechanical properties of the HCG

The mechanical properties of the HCG MEMS mirror, including spring constant, resonance frequency, effective mass and mechanical quality factor, can be characterized independently from the optomechanics phenomena, through either measurement or simulation. The spring constant k and the resonant frequency f_r can be simulated by finite element method (FEM). For the TE HCG MEMS mirror shown in Fig. 2 and Fig. 3, they are simulated to be 0.17 N/m and 146 kHz for the fundamental mode. The spring constant can also be extracted from the laser tuning curve shown in Eq. S4 and Fig. S2. It is extracted to be ~0.2 N/m, which is in good match with the FEM simulation.

III. Optical reflection properties of the HCG

The reflectivity of the HCG can be calculated by rigorous coupled wave analysis[36] or the analytical solution developed in our group[31]. Fig. S3 shows the HCG reflection spectrum, using the as-fabricated grating dimensions for a TE HCG obtained from the SEM image in Fig. 2c. Throughout the lasing range (1552 nm ~ 1575 nm as shown in Fig. 3a), the reflectivity is > 99%, and the reflection spectrum can be fitted by a quadratic equation:

$$R_1(\lambda) = R_{1p} + R_1'' \cdot (\lambda - \lambda_p)^2 \qquad \text{(S7)}$$

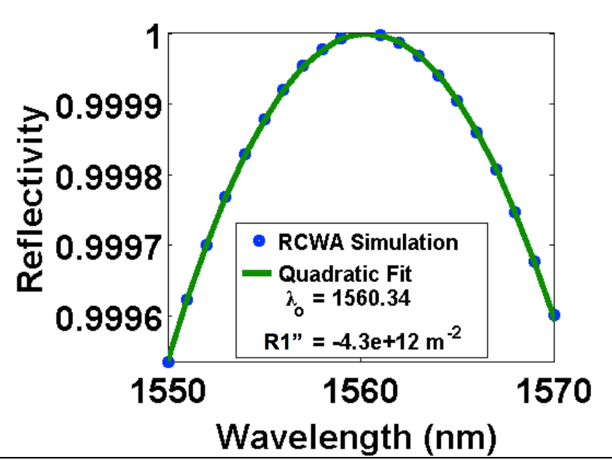

Figure S3 | A TE HCG mirror reflection spectrum calculated by RCWA (blue dots), curve fitted by a quadratic equation (green curve).

From Fig. S3, R_1'' (i.e.$d^2R_1/d\lambda^2$) is extracted to be -4.3x10^{12} m^{-2}, and the peak reflectivity R_{1p} is closed to 1. The RCWA-computed value is an excellent approximation to the actual HCG reflectivity, but due to sidewall roughness of the grating (on the order of SEM resolution of 10 nm), the overall HCG reflectivity could be slightly lower and less broadband. Thus, R_1'' would be larger in its absolute value, and R_{max} will be smaller. We relax R_1'' to be in the range of -5x10^{12} m^{-2} to -5x10^{13} m^{-2}, and R_{1p} in the range of 99.5%~99.9%. This would be a reasonable estimation for the actual mirror reflectivity. The dependence of HCG reflectivity versus wavelength (and thus HCG position) would be important in the laser optomechanical dynamics, as shown in Section IV.

IV. Theoretical analysis of the dynamics in laser optomechanics

IV.1: Overview of Rate Equations and Parameters

In laser optomechanics, two systems are coupled together. The first system is the laser itself, which involves the gain and the cavity. This can be described by the laser rate equations. The second system is the optomechanics, which involves the mechanical oscillator and the radiation pressure. This can be described by the motion equation of the harmonic oscillator. When implementing the laser optomechanics with an HCG VCSEL, we use the semiconductor laser rate equations to describe the laser behavior:

$$\frac{dN}{dt} = \frac{\eta I}{qV_a} - (AN + BN^2 + CN^3) - v_g g_o \frac{S}{1+\varepsilon S}(N - N_{tr}) \qquad \text{(S8)}$$

$$\frac{dS}{dt} = \Gamma v_g g_o \frac{S}{1+\varepsilon S}(N - N_{tr}) - \left(\alpha_i + \frac{1}{2L} ln\frac{1}{R_1(x)} + \frac{1}{2L} ln\frac{1}{R_2}\right) v_g S + \Gamma\beta B N^2 \qquad \text{(S9)}$$

The motion equation of the harmonic oscillator is described in Equation (S10),

$$m\ddot{x}(t) + b\dot{x}(t) + m\omega_o^2 x(t) = F_{OM}(x,t) = F_{RP}(x,t) + F_{PT}(x,t) \qquad \text{(S10)}$$

Table S1 describes the parameters used in Equation (S8)~(S10).

Table S1. Parameter definitions for laser rate equations

Symbol	Definition	(Typical) Values	Source
N	Carrier concentration	~10^{18} /cm^3	Derived parameter
N_{tr}	Carrier concentration at transparency condition	10^{17}~10^{18} /cm^3	Value from Ref. 37
A	Shockley-Read-Hall recombination coefficient	5×10^{7} /s	
B	Spontaneous emission coefficient	8×10^{-11} cm^3/s	
C	Auger recombination coefficient	3.5×10^{-30} cm^6/s	
I	Injection current	5~20 mA	Experiment conditions
η	Injection current quantum efficiency	0.9~0.95	Value from Ref. 37
S	Photon concentration	~10^{14}/cm^3	Derived parameter
g_o	Differential gain	1×10^{-16}~ 3×10^{-16} cm^2	Value from Ref. 37
ε	Gain suppression ratio	2×10^{-17} cm^3	
β	Rate of the spontaneous emission coupled into laser mode	10^{-4}	
α_i	Intrinsic loss	1~15 /cm	
v_g	Group velocity	8.5×10^{7}~ 1×10^{8} m/s	VCSEL epitaxial structure from design
V_a	Active region area	4~25 μm^3	
Γ	Mode confinement factor	0.01~0.04	VCSEL epitaxial structure from design and transmission matrix analysis
L	Effective cavity length	2~4 μm	
R_1	Top HCG reflectivity	0.99~1	HCG design and simulation with RCWA
R_1''	Second derivative of top HCG reflectivity to wavelength	-5×10^{12} ~$-5\times10^{13}/m^2$	
R_2	Bottom DBR reflectivity	0.995~1	VCSEL epitaxial structure
P_o	Laser output power	0.1~4 mW	Derived parameter; measurement
m	HCG mirror mass	100 ~300 pg	HCG design and FEM simulation
f_o	Intrinsic resonance frequency of the mechanical oscillator	0.1~6 MHz	FEM simulation; measurement
Q_m	Mechanical quality factor (Q)	10^3~10^4	Measurement
g_{om}	Optomechanics coupling coefficient	~32.2 GHz·rad/nm	VCSEL epitaxial structure from design; measurement
λ	Lasing wavelength	1540 ~ 1580 nm	Measurement
Note 1: Intrinsic resonance angular frequency of the mechanical oscillator $\omega_o = 2\pi f_o$<br>Note 2: mechanical damping coefficient $b = \omega_o m/Q_m$			

As described in the main body of the manuscript, the optomechanical forces F_{RP} and F_{PT} represent delayed responses to the change in position, and thus can be decomposed into a static effective spring force F_{OM}(x) and damping components:

$$m\ddot{x}(t) + \left[b + \frac{dF_{RP}}{dx}\cdot\tau_c + \frac{dF_{PT}}{dx}\cdot(\tau_c+\tau_{th})\right]\dot{x}(t) + [m\omega_o^2 x(t) - F_{OM}(x)] = 0 \quad \text{(S11)}$$

The radiation force F_{RP} depends directly on the intracavity circulating power P_c, and can be related to the photon density S using rate-equation parameters:

$$F_{RP} = \frac{2\mathrm{P_c}}{c} = \frac{SV_a/\Gamma}{\mathrm{c}L/v_g}\frac{hc}{\lambda} \quad \text{(S12)}$$

where c is the speed of light in air, and h is the Plank constant, and other variables are as described in Table 1.

While the laser is being continuously pumped, its radiation force F_{RP} pushes the HCG. At different HCG displacements, the circulating power inside the cavity changes due to the wavelength and mechanical deformation dependence of the HCG reflectivity. Both effects are treated as the change of the effective HCG reflectivity R_1. For the fundamental mechanical oscillation mode of the HCG, the HCG oscillates as a whole plane (Fig. 5a), the HCG reflectivity is dominantly dependent on wavelength. As shown in Fig. S3, the relationship between R_1 and λ can be described by a quadratic equation. Below, we provide a detailed analysis of the dynamics of radiation-pressure driven laser optomechanics and a treatment of photothermal dynamics.

IV.2: Detailed Analysis of Radiation-Pressure Based Laser Optomechanics

As described above, mechanical gain or damping terms of the form $\frac{dF}{dx} \cdot \tau(x)$ are added onto the original mechanical damping b for both F_{RP} and F_{PT}. If these terms provide a net damping $b_{eff} < 0$, instability and regenerative oscillation occur. Considering only radiation pressure dynamics, to obtain $\frac{dF_{RP}}{dx}$ and $\tau_c(x)$, we can calculate how the laser behaves dynamically in response to a perturbation of the HCG displacement x. This can be done by taking a differential analysis on Equation (S8), (S9) and (S11), similarly to the conventional differential analysis of small-signal current modulation of lasers. Due to a change of HCG displacement x, the resonant wavelength changes. This leads to a change of the HCG reflectivity (Fig. S3 and Eq. S7), and thus the circulating carrier concentration and photon concentration in the laser cavity. This then turns into a change of the radiation pressure force. The HCG reflection spectrum is of significance in this process.

We take the differential on Equation (S8), (S9) and (S11), and we assume the following forms of the small-signal response of carrier concentration $dN(t)$, photon concentration $dS(t)$, and radiation force $dF_{RP}(t)$ (temporarily discounting photothermal force) to a sinusoidal displacement modulation $dx(t)$:

$$dx(t) = x_1 e^{j\omega t}$$

$$dN(t) = N_1 e^{j\omega t}$$

$$dS(t) = S_1 e^{j\omega t}$$

$$dF_{RP}(t) = F_1 e^{j\omega t} \quad \text{(S13)}$$

where ω is the mirror oscillation angular frequency. We then have

$$\begin{bmatrix} \gamma_{NN} & \gamma_{NS} \\ \gamma_{SN} & \gamma_{SS} \end{bmatrix} \begin{bmatrix} N_1 \\ S_1 \end{bmatrix} = \begin{bmatrix} 0 \\ \gamma_x \cdot x_1 \end{bmatrix} \tag{S14}$$

$$F_1 = \frac{2}{\mathrm{c}} \left(\frac{V_a hc}{\Gamma\lambda} \frac{v_g}{2L} \right) S_1 - \frac{1}{\pi c} \left(\frac{S V_a h}{\Gamma} \frac{v_g}{2L} \right) g_{om} x_1 \tag{S15}$$

where

$$\gamma_{NN} = j\omega + A + 2BN + 3CN^2 + v_g g_0 \frac{S}{1 + \varepsilon S}$$

$$\gamma_{NS} = -v_g g_0 \frac{S}{(1 + \varepsilon S)^2} (N - N_{tr}) S + v_g g_0 \frac{1}{1 + \varepsilon S} (N - N_{tr})$$

$$\gamma_{SN} = \Gamma v_g g_0 \frac{S}{1 + \varepsilon S} + 2BN\Gamma\beta$$

$$\gamma_{SS} = -j\omega - \Gamma v_g g_0 \frac{\varepsilon}{(1+\varepsilon S)^2} (N - N_{tr}) S + \Gamma v_g g_0 \frac{1}{1+\varepsilon S} (N - N_{tr}) - \left(\alpha_i - \frac{ln R_1 R_2}{2L} \right) v_g$$

$$\gamma_x = -\frac{1}{2L} \frac{2R_1'' \frac{g_{om}\lambda^2}{2\pi c} \left(\frac{x g_{om}\lambda^2}{2\pi c} + \lambda_0 - \lambda_p \right)}{R_{1p} + R_1'' / \left[\frac{x g_{om}\lambda^2}{2\pi c} + \lambda_0 - \lambda_p \right]^2} v_g S \tag{S16}$$

dN/dx and dS/dx can then be solved with Equation (S16), and then dF/dx with Equation (S15). τ_c can be obtained using Equation (S17):

$$\tau_c = \frac{phase(dF/dx)}{\omega} \tag{S17}$$

Fig. S4 plots a typical frequency response of $|\frac{dF_{RP}}{dx}|$ and τ_c for an HCG VCSEL at a typical current value and mirror position. They show a transfer function with a resonance frequency at 1.84 GHz. For the fundamental oscillation mode (set at 125 kHz), $|\frac{dF_{RP}}{dx}|$, τ_c, and $|\frac{dF_{RP}}{dx}| \cdot \tau_c$ are calculated for each mirror displacement *x*, shown in Fig. 5d-e. To have the comparison with passive optomechanics, the differential analysis is done on the coupled equations based on those in Ref. 12. The simulation parameter is adapted from Ref. 12 as well, except that the optical Q of the cavity is set to be that defined by the mirror loss of the VCSEL, 17,600, and the input light power is set to be the same as the output light power of the VCSEL, 2 mW. Using the parameters defined in Ref. 12 (optical mode radius of the toroidal microcavity R = 29 μm, refractive index n_{eff} = 1.46, and number of optical resonance wavelengths along the cavity circumference N = 169, wavelength λ = 1550 nm), the dλ/dx (or d*ω*/d*x*) is comparable with that of the VCSEL.

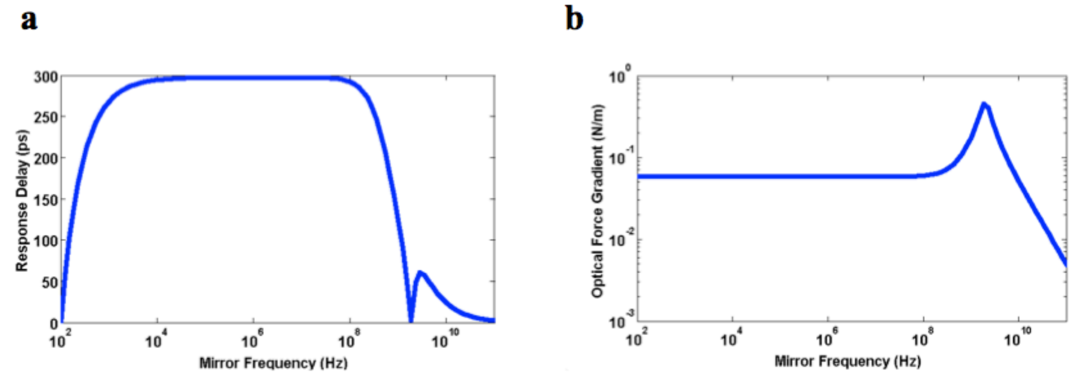

Figure S4 | Frequency dependence of (a) photon retardation time and (b) optical force gradient to a change of HCG displacement, using the same parameters as that in Fig. 5e.

The above analysis and simulation results shown in Fig. 5 show a much stronger light-matter interaction in laser optomechanics compared with passive cavity optomechanics. The response time τ_c is much longer in an active cavity than in an equivalent passive cavity with the same mirror-loss Q-factor, and optomechanical gain $\frac{dF_{RP}}{dx} \cdot \tau_c$ exists over a much broader position and wavelength range. To implement laser optomechanics, a laser with a mechanical oscillator is needed. Compared to macroscopic movable mirror reflectors, the HCG VCSEL offers ultra-light weight combined with high Q_m, producing a small mechanical damping coefficient b that can be overcome by the optomechanical gain $b_{RP} + b_{PT}$.

As is described in main manuscript, in passive cavity optomechanics, optical input power and detuning are the two control knobs to tune the optomechanical dynamics. In laser optomechanics, these correspond to the laser pumping strength and the cavity length, and specifically, the laser drive current and the tuning voltage of the HCG MEMS structure. Here, we further explain the influence of laser drive current on optomechanical dynamics.

With increasing drive current, the output power and the corresponding circulating power increases, as shown experimentally in the measured L-I curve, Fig. S5d. Likewise, the response time of the photon number to changes in mirror position shown in Fig. S5a decreases, as is predicted by the semiconductor rate equations due to decreasing carrier lifetime. This effect would tend to weaken the optomechanical negative damping. On the other hand, in Fig. S5b, the derivative of radiation pressure with respect to mirror position increases in magnitude, simply due to the increase in radiation pressure force at high currents due to the increased power shown in Fig. S5d. Based on the mechanism for optomechanical negative damping described in Part IV, the relevant quantity is the product of these two effects (i.e. anti-damping or optomechanical gain), shown in Fig. S5c. This quantity increases with applied current, predicting an increased wavelength swept range with increased current, as is reported experimentally in Fig. 3c.

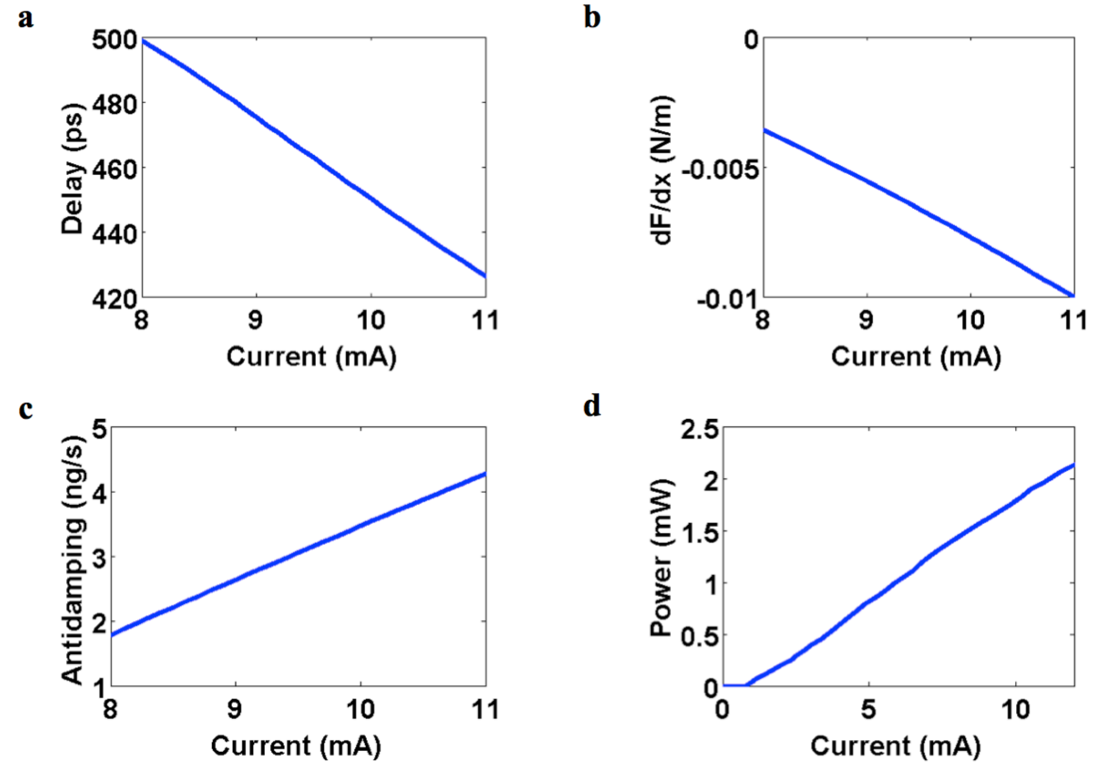

Figure S5 | Photon retardation, the gradient of the radiation force, overall mechanical gain versus drive current, and output power. a, Delay of the response of photon force to change in mirror position. This delay results from the effective photon lifetime in the rate equations, and is crucial in the damping cancellation effect of radiation pressure. As the semiconductor rate equations predict, increasing current reduces the photon lifetime. **b,** The gradient of the radiation force versus mirror position is negative, as is required for a heating rather than cooling optomechanical effect. Its absolute value increases with current due to increased photon population and thus radiation pressure. **c,** The overall anti-damping (or optomechanical gain) increases with current. This explains the effect of increased current in Fig. 3c. **d,** Experimental optical power vs. current (L-I) data for a representative 1.55μm HCG VCSEL.

IV.3: Photothermal Dynamics and Comparison

In this section, we evaluate the photothermal effect in the HCG VCSEL laser optomechanics. First, we calculate the optical power being absorbed in the top mirror, i.e. the InP HCG layer. The lasing wavelength is 1550 nm, corresponding to photon energy of 0.8 eV, well below the bandgap of InP. Thus, the only optical absorption mechanism is free carrier absorption of 44 cm^{-1} for p-InP at p = 3e18 cm^{-3} doping level[38]. At an output power of 1.7 mW, the circulating cavity power is ~850 mW using a top mirror reflectivity of 99.8%. The duty cycle of the grating gives a transverse confinement factor of 32%. Using a mirror reflectivity of 99.8%, as well as group velocity and effective cavity length values from Table S1, this gives an absorbed power of 440 μW in the HCG.

Using this value of absorbed power in the HCG, we perform FEM thermal time transient modeling to determine the photothermal force response time. With standard

values for InP thermal conductivity and heat capacity, and a test heat source of 2 μW in the HCG bars representing absorption, time-domain results shown in Fig. S6 indicate that the thermal time constant of the grating structure is 19 μs, limited by the small thermal conductivity down the long, thin MEMS support arms. This time constant corresponds to a frequency of 53 kHz, which is significantly lower than the range of self-oscillation frequencies reported in this paper (120 kHz - 3.5 MHz). This time constant adds a low-pass response into the photothermal dynamics, as discussed below.

Lastly, we use FEM steady-state thermal modeling to show the 3D distribution of temperature and physical warping of the MEMS structure due to photothermal effects, with a 440 μW heat source applied on the bottom of the bars, and modeling thermal expansion of the InP. In Fig. S7a, we show the 2D distribution of temperature along the surface of the MEMS structure. It shows a 135 K warming due to 440 μW found above, and that the temperature change in the MEMS support arms is significantly smaller (< 80 K). The simulated thermal expansion is illustrated in Fig. S7b, showing a static vertical displacement of the grating due to thermal expansion of Δx_{PT} = 384 pm, or F_{PT} = 2.2 nN given k_{spr} = 0.17 N/m. The amplitude induced by sinusoidally modulated light intensity at the mechanical resonance frequency ω_o, as is produced in self-oscillation, can be related to the static displacement, with the optical Q and a low-pass filter response[21]:

$$x_{PT}(\omega_o) = Q_m \frac{\omega_o \tau}{1+(\omega_o \tau)^2} \frac{x_{PT,DC}}{P_{DC}} P(\omega_o) \qquad \text{(S18)}$$

With the static thermal displacement of 384 pm, and for complete on-off modulation of light power $P(\omega_o)$ = 1.7 mW, this results in a displacement oscillation amplitude $x_{PT}(\omega_o)$ = 153 nm. Thus, given the available device parameters, the photothermal force accounts for a significant portion of the energy transfer from light into the mechanical mode, but cannot account for the observed self-oscillation amplitude (~600 nm) without radiation-pressure forces as well.

Applying this simple analysis to radiation-pressure dynamics, which does not have the same low-pass filter effects,

$$x_{RP}(\omega_o) = Q_m \frac{x_{RP,DC}}{P_{DC}} P(\omega_o) = Q_m \frac{2}{c k_s} P(\omega_o) \qquad \text{(S19)}$$

the observed modulation $P(\omega_o)$ = 1.7 mW provides $x_{RP}(\omega_o)$ = 850 nm, illustrating that radiation pressure can be the main source of the observed optomechanical oscillation.

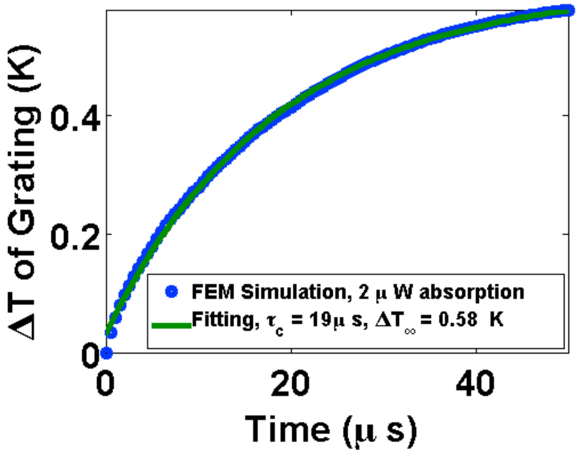

Figure S6 | Thermal transient curve of the HCG simulated in FEM. Under test heat source of 2 μW in the HCG bars, the thermal time constant of the grating structure is fitted to be 19 μs.

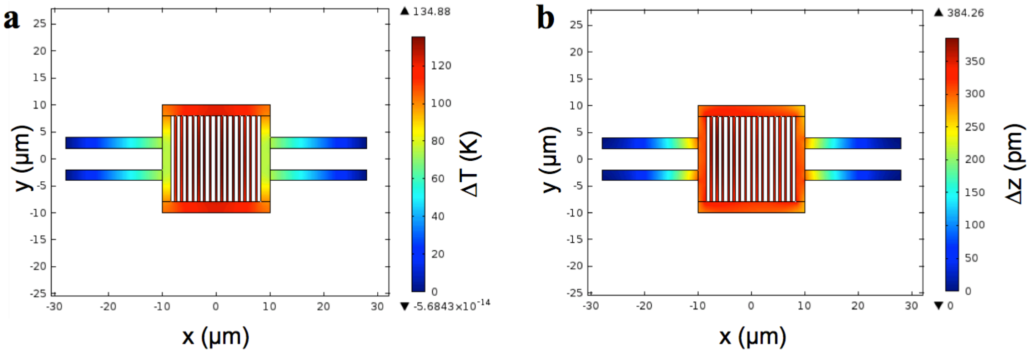

Figure S7 | Thermal distortion simulation of the HCG MEMS structure using FEM. A 440 μW heat source is applied on the bottom of the grating bars made of InP. **a**, 2D distribution of temperature along the surface of the MEMS structure showing a maximum ΔT = 135 K. **b**, thermal expansion of the HGC MEMS structure due to 440 μW heat absorption at the bottom of the bars showing a maximum Δz = 384 pm. Note, this Δz is labelled as Δx_{PT} in the text.

V. Multi-mechanical-oscillation-mode in laser optomechanics

Regenerative oscillation with more than one mechanical mode can exist in HCG VCSEL. Fig. 4 shows the two different states of mechanical regenerative oscillation, one in fundamental mode, and the other in a high-order mechanical mode. At certain values of electrical input parameters (laser current and MEMS voltage), both modes can self-oscillate simultaneously. Fig. S8 shows the RF spectrum and the time-domain optical power of the laser output. The 353.6 kHz and 5.636 MHz RF tone are the oscillation frequency of these modes. Their frequency can be different from that in the single mode state, due to the optical spring effect. Beating between the two modes is seen in the RF spectrum. The time-domain laser output power waveform is a superimposition of that from the pure fundamental mode state and pure high order mode state.

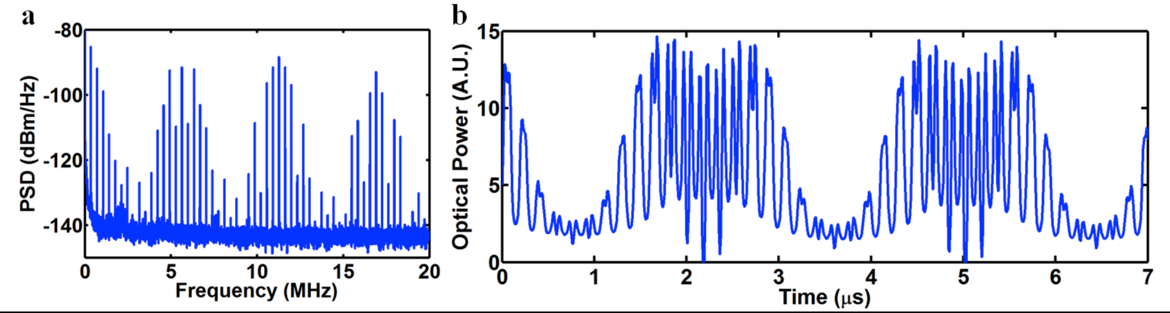

Figure S8 | Regenerative oscillation of the HCG VCSEL with multi-mechanical-oscillation-mode. a, RF spectrum of the laser optical output power. **b**, Laser optical output power in time-domain. The threshold current of the laser is 10.1 mA at room temperature. The laser current and tuning voltage is 18 mA, 7.04 V respectively.

VI. Characterization of oscillation frequency and amplitude from SEM image

Fig. 2d shows the SEM image of the HCG oscillating at fundamental mechanical mode. In the SEM image, the electron beam scans along the grating bars. Due to a fast scanning rate, stroboscopic effect can be visualized on the grating bars, i.e. the aliasing period structures along the grating bars. This can be used to calculate the mechanical oscillation frequency and amplitude. The SEM image has a pixel size of 1024 by 768. The image dwell time per pixel is 400 ns. In Fig. 2d, the horizontal aliasing period is counted to be ~19.1 pixels. Thus the oscillating frequency is calculated to be $1/(19.1\times400\text{ns}) = 131\,\text{kHz}$. In this image, the peak-peak amplitude of vertical MEMS oscillation is determined to be ~595 nm based on scaled comparison with the known 1070 nm HCG bar period, accounting for the view elevation angle of 45°.

VII. Material system for III-V active optomechanical devices

The high contrast grating VCSEL used for laser optomechanics represents a unique marriage between optoelectronics and optomechanics. With its unique combination of light weight and high reflectivity, HCG has made it possible to exhibit laser optomechanics observed in this paper. In this paper, the high contrast grating VCSEL emits light in 1.55 μm wavelength using InP/AlGaInAs material system. Another commonly used material system is GaAs/AlGaAs system producing laser light at a wavelength of 0.85 μm . We have also observed laser optomechanical self-oscillation in a high contrast grating VCSEL using GaAs as gain material and AlGaAs as high contrast grating. In both examples, the high contrast grating is optically transparent to the laser light, avoiding photothermal effects. This demonstrates that versatile material systems can be used in laser optomechanics. These III-V material systems will play an important role in the on-chip integration between optoelectronics and optomechanics.

References

36. Moharam, M. G. & Gaylord, T. K. Rigorous coupled wave analysis of planar grating diffraction. *J. Opt. Soc. Am.* **71**, 811 (1981).

37. Coldren, L. A., Corzine, S. W. & Masanovic, M. L. Diode lasers and photonic integrated circuits, second edition. John Wiley & Sons, Inc. 2012.

38. Gonzalez, L. P., Murray, J. M., Krishnamurthy, S., and Guha, S. Wavelength dependence of two photon and free carrier absorptions in InP. *Opt. Express* **11**, 8741-8748 (2009).
